# Association of the controlling nutritional status score with all-cause mortality and cancer mortality risk in patients with type 2 diabetes: NHANES 1999–2018

**DOI:** 10.1186/s13098-023-01138-2

**Published:** 2023-08-21

**Authors:** Dikang Pan, Julong Guo, Zhixiang Su, Jingyu Wang, Sensen Wu, Jianming Guo, Yongquan Gu

**Affiliations:** 1 Xuanwu Hospital, Capital Medical University, Beijing, China; 2 Renal Division, Peking University First Hospital, Beijing, China

**Keywords:** National Health and Nutrition Examination Survey, Controlling Nutritional Status score, cancer mortality, All-cause mortality

## Abstract

**Objective:**

There are studies on the nutritional status of type 2 diabetes (T2D), but there are no large cohort studies on the prognosis of Controlling Nutritional Status (CONUT) score for T2D. The aim of this study was to examine the association between CONUT score and all-cause mortality as well as cancer mortality in adults with T2D.

**Methods:**

For this study, we analyzed a total of 3763 adult patients with T2D who were part of the National Health and Nutrition Examination Survey (NHANES) from 1999 to 2018. Mortality outcomes were determined by linking to the National Death Index records as of December 31, 2019. Cox proportional risk models were used to estimate risk ratios (HRs) and 95% confidence intervals (CIs) for all-cause and cancer deaths.

**Results:**

During the mean follow-up of 8.17 years, there were 823 deaths from all causes and 155 deaths from cancer. After adjusting for multiple variables, the risk of all-cause mortality was higher in patients with a Mild (CONUT score ≥ 2), compared with patients with a Normal (CONUT score of 0–1). All-cause mortality risk was 39% higher, and cancer mortality risk was 45% higher. Consistent results were observed when stratified by age, sex, race, BMI, smoking status, and glycated hemoglobin levels.

**Conclusions:**

In a nationally representative sample of American adults with T2D, we found an association between CONUT score and all-cause mortality and cancer mortality.

**Supplementary Information:**

The online version contains supplementary material available at 10.1186/s13098-023-01138-2.

## Introduction

Currently, there are approximately 537 million adults worldwide with diabetes, of whom over 90% suffer from type 2 diabetes (T2D). This contributes to a prevalence rate that has reached as high as 10.5% among adults [1]. Individuals with diabetes face a 2–4 times greater risk of cardiovascular disease (CVD) and mortality compared to non-diabetics [2]. Therefore, it is crucial to identify prognostic factors that can potentially prevent or delay diabetic complications and death.

Malnutrition is typically defined as the presence of a low body mass index (BMI) and low serum albumin levels [3]. It is associated with a variety of metabolic abnormalities, including steatosis, increased lipolysis and fatty acid oxidation, decreased circulating amino acids, reduced peroxisome number and function, and impaired mitochondrial function [4]. Malnutrition can also lead to immune dysfunction and increased mortality from infection [5–7]. Acute or chronic diseases and their therapeutic interventions may also lead to an increase in malnutrition, mainly due to altered metabolism [8]. The high prevalence of diabetes-related complications and comorbidities may further compromise nutritional status, and malnutrition can lead to impaired muscle function and wound healing, reduced bone mass, immune dysfunction, and decreased systemic function [9, 10]. Various scores are available to reflect human nutritional status, including the prognostic nutritional index (PNI), the controlled nutritional status score (CONUT score), and the nutritional risk index (NRI) [11–13].

In this paper, we hypothesize that the CONUT score is closely related to the development of T2D. To address this question, we aim to prospectively investigate the relationship between the CONUT score and all-cause mortality, as well as cancer mortality, in a nationally representative sample of adult patients with diabetes in the United States.

## Methods

### Study population

The National Health and Nutrition Examination Survey (NHANES) is an ongoing research project that provides estimates of the population’s nutrition and health status in the United States. This survey uses a stratified, multi-stage probability design to recruit a representative sample of the American population. Data is gathered through structured interviews with individuals at home, health screenings at mobile health screening centers, and laboratory sample analysis [9].

**Fig. 1:**
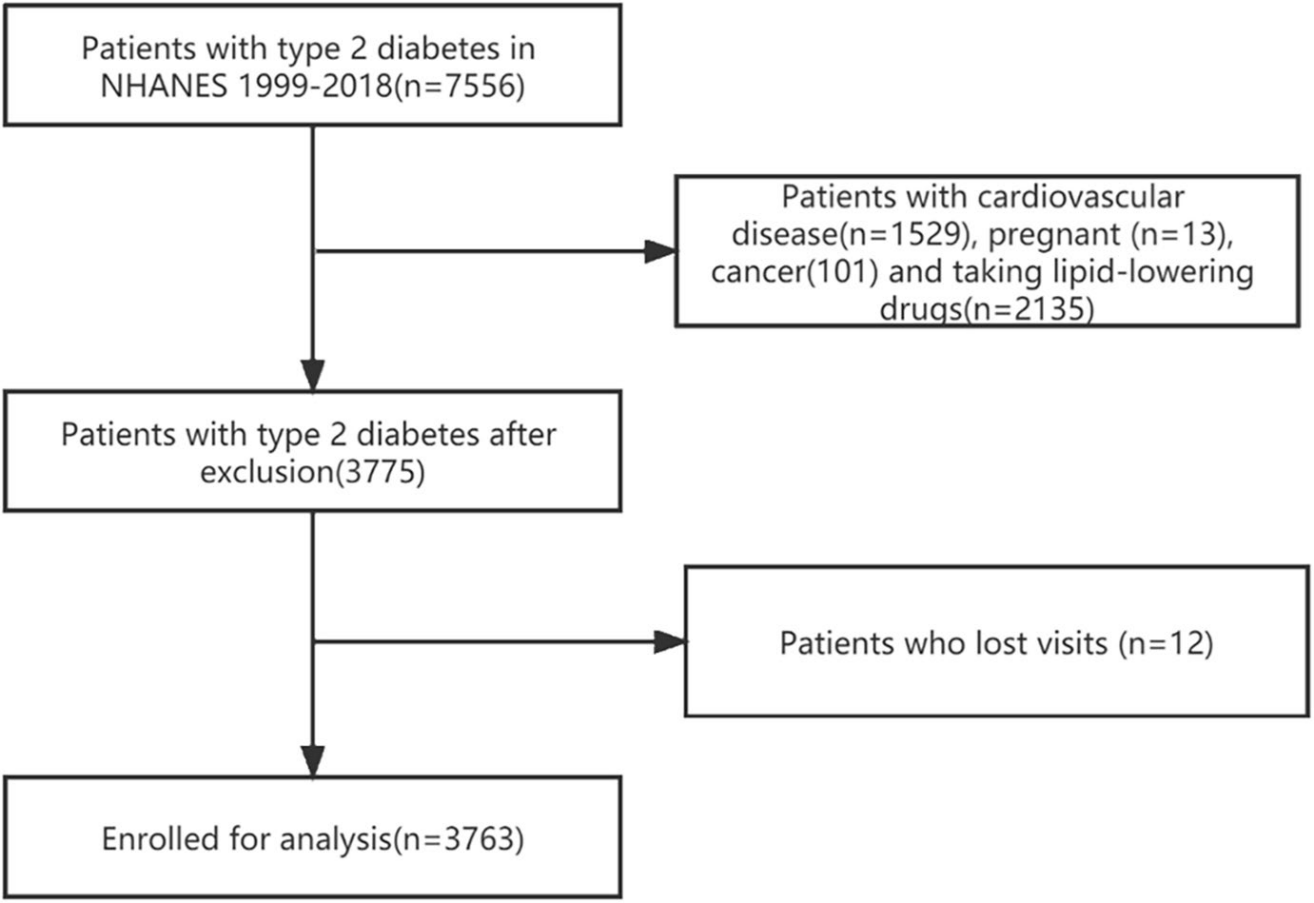
Flowchart describing the sample exclusion criteria used in this study which uses data from NHANES (1999–2018)

For this study, we analyzed data from NHANES 1999–2018, which provided information on s which provided information on CONUT score. We only included patients who were over 20 years old and had been diagnosed with T2D, resulting in a study sample of 7556 subjects. T2D was defined as a diagnosed case of diabetes mellitus with insulin or oral hypoglycemic agents and fasting glucose levels above 7.0 mmol/L (126 mg/dL) or glycated hemoglobin A1c (HbA1c) levels above 6.5%. We excluded participants who were pregnant (n = 16), had cardiovascular diseases (n = 1529), cancer (n = 101), taking lipid-lowering medication (n = 2135) and missed visits (n = 12) resulting in a final representative analysis of 3763 T2D patients (Fig. 1).

### Evaluation of CONUT scores

The CONUT score was developed in 2005 by Ulibarri et al. [14] as a tool for screening nutritional status in hospitalized patients. The score is based on three parameters: albumin, total cholesterol, and lymphocyte. A score of 0 to 1 indicates normal nutritional status, while a score of 2–4 indicates mild malnutrition, 5–8 for moderate malnutrition, and 9–12 for severe malnutrition. Participants were divided into nutritional normal group (CONUT:0–1) and malnutrition group (CONUT ≥ 2).

### Determination of mortality

All-cause mortality and cancer mortality were determined through documentation obtained from the National Death Index as of December 31, 2019. To determine disease-specific deaths, we used the International Classification of Diseases (ICD)-10. Deaths related to cancer were defined using ICD-10 codes C00-C97. In total, there were 1,248 recorded deaths, including 227 deaths due to cancer.

### Assessment of covariates

Information on participants’ socio-demographic characteristics, smoking status, alcohol consumption, duration of diabetes, use of diabetes medication, and hypertension was collected using a standardized questionnaire. Participants who had smoked fewer than 100 cigarettes during their lifetime were classified as non-smokers, while those who had smoked more than 100 cigarettes in the past but had not quit were defined as current smokers. Former smokers were those who had smoked more than 100 cigarettes in the past, but had quit. Drinking status was categorized into three levels: non-drinker, low to moderate drinker (less than 2 drinks per day for men and less than 1 drink per day for women), and heavy drinker (2 or more drinks per day for men and 1 or more drink per day for women). Race/ethnicity was classified as non-Hispanic white or other. Educational attainment was categorized as less than high school, high school or equivalent, or college or higher. Poverty income ratio (PIR) scores were defined as 0-1.3, 1.3–3.49, and greater than or equal to 3.5. BMI was calculated as weight divided by height squared (kg/m^2^), and glycated hemoglobin was also assessed as previously described [15].

### Statistical analysis

Due to the complex sampling design of the NHANES database, analysis of the data required consideration of sample weights, clustering, and stratified analysis. Normally distributed data were expressed as standard deviations, while non-normally distributed data were expressed as medians and interquartile ranges. Categorical variables were expressed as percentages and analyzed using chi-square tests, while quartiles of CONUT score levels were identified based on the distribution of the study population. One-way ANOVA tests (for continuous variables with normal distribution), Kruskal-Wallis tests (for continuous variables with non-normal distribution), and chi-square tests (for categorical variables) were used to compare differences between the four groups. Cox proportional hazards regression was used to estimate risk ratios (HRs) and 95% confidence intervals (CIs) for cardiovascular disease mortality associated with CONUT score. In the multivariate model, multiple imputation was used to deal with data with missing covariates. It is well known that multiple imputation methods provide better results than the complete data method of analysis. Follow-up was calculated as the time interval between the NHANES interview date and either the date of death or the end date of follow-up (December 31, 2019), whichever occurred first.

To investigate the dose-response relationship between CONUT score level and all-cause mortality and cancer mortality, we used restricted cubic spline (RCS) analysis. In our multivariable model, we adjusted for age (in years), sex (male or female), and race/ethnicity (non-Hispanic white or other) in model 1. In model 2, we further adjusted for BMI (in kg/m2) using the categories of 25.0-29.9 and ≥ 30.0, education level (below high school, high school or equivalent, or college or above), PIR(< 1.3, 1.3–3.49, and ≥ 3.5), smoking status (never, former, or current smoker), and drinking status (none, low to moderate, or heavy drinkers). In model 3, we further adjusted for diabetes duration (in years), diabetes medication use (none, oral medication only, insulin only, or both insulin and medication), HbA1c (≥ 7.0% or < 7.0%), and hypertension (yes or no).

We also conducted several sensitivity analyses to assess the robustness of our findings. In stratified analyses, we divided participants based on age (≤ 60 or > 60 years), sex (male or female), race/ethnicity (non-Hispanic white or other), smoking status (current or never/past), BMI (≥ 30 or < 30 kg/m2), and HbA1c (≥ 7 or < 7%). We assessed the significance of the interaction by examining the P-value of the product term between the CONUT score level and the stratified variables.

To address potential reverse causality bias, we excluded participants who had died 2 years prior to follow-up (n = 392). Additionally, we combined mild and moderately severe patients in the CONUT score and reanalyzed the results. Furthermore, we included C-reactive protein (CRP) levels, lipid profiles (including triglycerides, total cholesterol, low-density lipoprotein, and high-density lipoprotein cholesterol), as well as indicators of kidney function (creatinine and uric acid levels) and liver function (aspartate aminotransferase and alanine aminotransferase levels) as additional adjustments to investigate the potential influence of inflammation, lipid levels, and liver and kidney indices on the observed associations.

Given the non-normal distribution of the CONUT score, we used an unadjusted Spearman correlation coefficient for the correlation analysis at baseline. We also plotted Kaplan-Meier survival curves to examine the association between the CONUT score and both all-cause death and cancer death. Finally, we conducted a restricted three-sample analysis by incorporating relevant hematological indicators into the baseline data to further explore the sensitivity of our findings.

## Results

In the study of 3763 individuals with T2D (mean age: 56.12 years; 50.0% male), we observed 823 all-cause deaths and 155 cancer-related deaths over an average follow-up period of 98.56 months. Table 1 displays the baseline characteristics of participants based on CONUT score severity and relevant variables. In comparison to individuals with normal nutrition (n = 3087), those with mild malnutrition (n = 676) exhibited certain differences. They tended to be older, with a mean age of 59.50 years compared to 56.11 years. Additionally, there was a higher proportion of males in the mild malnutrition group, with 410 individuals (60.7%) compared to 1472 individuals (47.7%). Furthermore, the mild malnutrition group had a lower body weight, with 136 individuals (20.1%) falling within the BMI range of 18.5-24.99, whereas 442 individuals (44.3%) fell within that range in the normal nutrition group. The mild malnutrition group also had a longer duration of diabetes (> 10 years), with 204 individuals (30.2%) in contrast to 611 individuals (19.8%) in the normal nutrition group. Lastly, there was a higher prevalence of hypertension in the mild malnutrition group, with 355 individuals (52.2%) compared to 1488 individuals (48.2%) in the normal nutrition group.

**Table 1:** Baseline characteristics of patients with T2D in CONUT score and NHANES, 1999–2018

Characteristic	Total				P value
		Normal nutrition (3087)	Malnutrition (676)		
Age(years)	56.72 ± 14.93	56.11 ± 14.63	59.50 ± 15.95		< 0.001
Male(n,%)	1882(50.0%)	1472(47.7)	410(60.7)		< 0.001
Hypertension (n,%)	1841(48.9%)	1488(48.2)	353(52.2)		0.002
**Education level (n,%)**					0.088
Less than high school	1414(37.6%)	1183(38.3)	231(34.2)		
High school diploma or GED	903(24.0)	740(24.0)	163(24.1)		
More than high school	1446(38.4%)	1164(37.7)	282(41.7)		
**PIR (n,%)**					0.400
< 1.3	1224(32.5)	1001(32.4)	223(33.0)		
1.3–3.49	1715(45.6)	1421(46.0)	294(43.5)		
≥ 3.5	824(21.9)	665(21.5)	159(23.5)		
Non-Hispanic White	1181(31.4)	961(31.1)	220(32.5)		0.473
**BMI, kg/m** ^**2**^					< 0.001
<18.5	23(0.6)	15(0.5)	8(1.2)		
18.5-24.99	578(15.4)	442(44.3)	136(20.1)		
25.0-29.99	1146(30.5)	935(30.3)	211(31.2)		
≥30.0	2016(53.6)	1695(54.9)	321(47.5)		
**Drinking status**					0.491
Nondrinker	2012(53.5)	1643(53.2)	369(54.6)		
Low-to-moderate drinker	374(10.2)	310(10.0)	74(10.9)		
Heavy drinker	1367(36.3)	1134(36.7)	233(34.5)		
**Smoking status**					0.239
Never smoker	1880(50.0)	1543(50.0)	337(49.9)		
Ever smoker	1248(33.2)	1010(32.7)	238(35.2)		
Current smoker	635(16.9)	534(17.3)	101(14.9)		
**Duration of diabetes**					< 0.001
< 3years	709(18.8)	589(19.1)	120(17.8)		
3–10 years	753(20.0)	599(19.4)	154(22.8)		
> 10 years	815(21.7)	611(19.8)	204(30.2)		
**Medication use**					0.595
No insulin or pills	1440(38.3)	1186(38.4)	254(37.6)		
Only diabetes pills	1597(42.4)	1318(42.7)	279(41.3)		
Only insulin	360(9.6)	288(9.3)	72(10.7)		
Diabetes pills and insulin	366(9.7)	295(9.6)	71(10.5)		
HbA1c (< 7.0%)	2338(62.1)	1910(62.0)	428(63.3)		0.509

Data are numbers (percentages) unless otherwise noted. All estimates include a complex survey design

### CONUT score and all-cause mortality

**Fig. 2:**
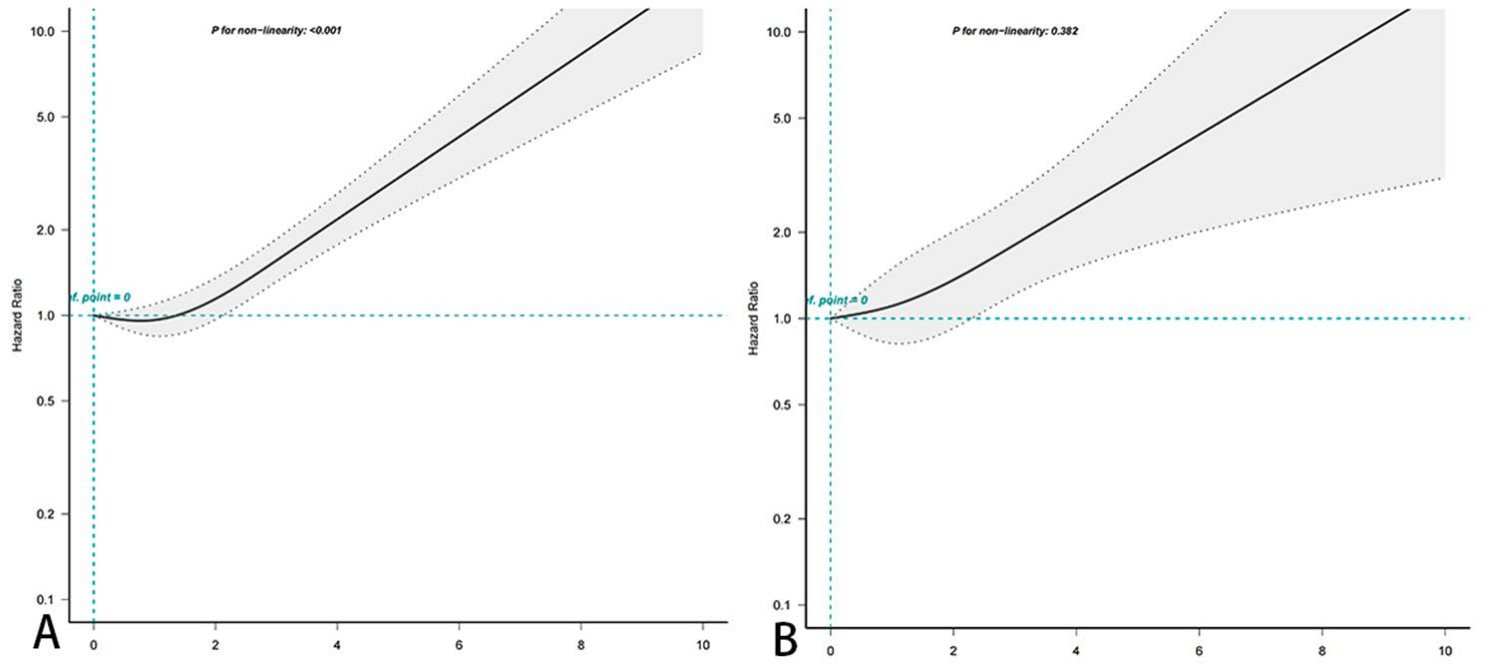
Association between CONUT score and all-cause **(A)** and cancer mortality **(B)** in patients with diabetes in the NHANES study from 1999–2018. Hazard ratios (solid lines) and 95% ci (shaded areas) according to age (continuous), sex (male or female), race and ethnicity (non-Hispanic white or other), BMI (< 18.5, 18.5-24.99, 25.0–29.99, or ≥ 30.0 kg/m^2^), education level (< high school, high school or equivalent, or above high school), PIR (lower, normal, or higher), smoking status (never, past, or current), alcohol use (none, low to moderate, or heavy), duration of diabetes (< 3years, 3–10 years, > 10 years), medication use (no insulin or pills, only diabetes pills, only insulin, diabetes pills and insulin) and HbA1c (< 7.0%,≥7.0%) to adjust. P < 0.001 for all-cause mortality and P-value of 0.096 for cancer mortality

A non-linear association was observed between the CONUT score and all-cause mortality (P = 0.001 for non-linearity) as depicted in Fig. 2A. Upon adjusting for various factors in the multivariate analysis, which included age, sex, race, BMI, duration of diabetes, diabetes medication use, and hypertensive disease, the malnutrition group remained significantly associated with an increased risk of all-cause mortality, as shown in Table 2. The HRs for all-cause mortality were 1.52 (95% CI, 1.30–1.79) for the mild malnutrition group when compared to the normal nutrition group. Since the CONUT score was found to be nonlinear in Fig. 2A and the risk of a 0 score was slightly higher than a 1 score, we performed an analysis, which showed no statistically significant difference between a CONUT ≥ 1 and a CONUT < 1 (eTable 4 in the Supplement).

**Table 2:** HR (95%CIs) for all-cause mortality and cancer mortality associated with serum CONUT Score in patients with diabetes mellitus in the NHANES study, 1999–2018

CONUT score			
	Normal nutrition	Malnutrition	P
All-cause mortality death, No/total No	611/3087	212/676	
Model 1	1.00	1.40(1.23,1.59)	< 0.001
Model 2	1.00	1.42(1.25,1.61)	< 0.001
Model 3	1.00	1.39(1.23,1.59)	< 0.001
Cancer mortality death, No/total No	115/3087	40/676	
Model 1	1.00	1.52(1.04,2.20)	0.035
Model 2	1.00	1.49(1.03,2.15)	0.026
Model 3	1.00	1.45(1.01,2.10)	0.040

Model 1: Adjusted for age (continuous), sex (male or female), and race/ethnicity (non-Hispanic white, non-Hispanic black, Mexican American, or other). Model 2: Further adjusted (from model 1) for BMI (< 18.5, 18.5-24.99, 25.0–29.99, or ≥ 30.0 kg/m2), education level (below high school, high school or equivalent, or college or above), PIR (< 1.3,1.3–3.49, or ≥ 3.5), drinking status (non-drinker, low to moderate drinker, or heavy drinker), and Smoking status (never smoker, past smoker, or current smoker). Model 3: Further adjustment from model 2 was made for duration of diabetes (≤ 3year,3–10year, or > 10 years), diabetes medication use (none, oral medication only, insulin, or other), glycated hemoglobin (< 7.0% or ≥ 7.0%), and self-reported hypertension (yes or no)

### CONUT score and cancer mortality

No nonlinear association was observed between CONUT score and cancer mortality (P = 0.382 for nonlinearity) as shown in Fig. 2B. Afterwards, multivariate analysis was conducted to adjust for various factors including age, sex, race, BMI, duration of diabetes, diabetes medication use, and hypertensive disease. The results indicated that the HRs for cancer mortality in the malnutrition group were 1.45 (95% CI, 1.01,2.10) compared to the normal nutrition group.

### Stratified and sensitivity analyses

When analyses were stratified by age (≤ 60 or > 60 years), sex (male or female), race/ethnicity (non-Hispanic white or other), BMI (< 30 or ≥ 30 kg/m^2^), smoking status (current or never/past), and HbA1c (< 7 or ≥ 7%) (eTable 2 and eTable 3 in the supplement). After several tests, no significant interactions were found for all-cause mortality and cancer mortality.

In addition to this, several sensitivity analyses were performed to confirm the stability of the results, which were generally robust when excluding participants who died within 2 years of follow-up (eTable 4 in the Supplement). The same results were observed if the malnutrition group was divided into mild and moderate to severe malnutrition groups, adjusted for multiple factors(eTable 5 in the Supplement), and we also adjusted for relevant serological indicators (eTable 6 in the Supplement), which showed no substantial change in all-cause mortality, adjusting for CRP (model 2 ), lipid markers (triglycerides, total cholesterol, LDL and HDL, model 3), liver markers (alanine aminotransferase, aspartate aminotransferase, model 4) and renal markers (creatinine and uric acid, model 5) as evidenced by the correlations found. In terms of cancer mortality, after adjusting for CRP and lipid indices, no significant correlations were found (P = 0.054 and P = 0.678), suggesting that CONUT score may act on T2D through the inflammatory pathway. further analysis using Spearman correlations showed that CONUT score were associated with age, glycated hemoglobin, triglycerides, total cholesterol, low density and alanine aminotransferase (eTable 7 in the Supplement). We plotted the survival curves of CONUT score with all-cause death and cancer death, and the results showed that all were statistically significant (eFigure1 in the Supplement, P < 0.001). Notably, there was no substantial change in RCS when all values were included (eFigure2 in the Supplement).

## Discussion

In this large prospective cohort study of US adults with diabetes, we found a significant association between participant CONUT score levels and all-cause mortality and cancer mortality. After multivariate adjustment, a high CONUT score (≥ 2) was associated with increased all-cause mortality (P < 0.001) and a CONUT score of ≥ 5 was associated with increased cancer mortality (P = 0.007), and various stratified and sensitivity analyses indicated that our findings were reliable.

T2D is the most common type of diabetes mellitus in adults, for which there is no cure, and as a lifelong metabolic disease, lifestyle interventions while receiving oral hypoglycemic agents and insulin therapy can help achieve remission of T2D (blood glucose can still be in compliance or normal without the use of hypoglycemic agents), which is important to improve the psychological, physical, social, and economic stress of patients [16, 17]. Malnutrition refers to the situation of insufficient, excessive, or imbalanced intake of energy and/or nutrients. Patients with T2D are prone to malnutrition due to gastrointestinal disorders that affect digestion and absorption, massive loss of nutrients from the body due to polyuria, and insufficient intake of micronutrients due to unbalanced and unreasonable diets, which will not only affect glycemic control, but also aggravate vascular lesions due to chronic inflammatory reactions during malnutrition. This will not only affect glycemic control, but also aggravate vascular lesions due to chronic inflammation during malnutrition, leading to a significantly higher risk of mortality [18, 19]. Therefore, medical nutrition therapy recommends nutritional interventions for patients with T2D to promote glycemic control and improve prognosis [20].

There is no gold standard for clinical evaluation of malnutrition, and commonly used indicators to evaluate nutritional status include BMI and albumin [3], but they do not adequately reflect the nutritional status of patients. Tools such as the nutritional risk screening 2002, the subjective global assessment method, and micro-nutritional assessment have some value in evaluating nutritional status, but the assessment process relies on medical specialty examinations or subjective patient recall, which may lead to biased results. The CONUT score was proposed by Ulíbarri et al. [14] in 2005 for early screening and continuous monitoring of nutritional status of hospitalized patients. In recent years, several studies have shown the advantages of the CONUT score in evaluating the nutritional status of inpatients with cardiovascular diseases, malignant neoplasms, and other diseases in a simple and objective way [21–23].

In our study of T2D patients, the percentage of CONUT malnourished patients was 21%. Furthermore, 1.0% of patients were classified as moderately to severely malnourished according to assessment. The CONUT assessment of the nutritional status of T2D patients suggests that malnutrition is quite common in these patients. On the one hand, this may be due to the interrelationship between T2D and inflammation [4, 24, 25]. Previous studies have reported that diabetic patients have higher levels of pro-inflammatory cytokines and that activation of inflammatory pathways may increase catabolic demand, leading to malnutrition [26]. On the other hand, vitamin D deficiency, which is common in malnourished patients, ensures the maintenance of normal low physiological levels of Ca^2+^ and reactive oxygen species in the body [27, 28], and may also play a role by reducing inflammation and contributing to the control of insulin resistance, which is a major trigger of diabetes [29].

In the results of the subgroup analysis of cancer mortality, we found several interesting phenomena. First, in the age subgroup results, we found that malnourished T2D patients > 60 years of age were more likely to have cancer mortality than those ≤ 60 years of age. With the coexistence of multiple diseases and the progressive aging of physiological functions and tissues and organs in the elderly, the blow of malnutrition seems to be more fatal, and it is important to assess the degree of malnutrition in elderly T2D patients and to take measures to improve it [30]. Second, in the gender subgroup, male T2D patients diagnosed as malnourished according to CONUT have a higher likelihood of cancer mortality than female T2D patients, and the 2020 World Health Organization reported that in 2020, there were 10,065,305 new cases of cancer with a crude incidence rate of 256.1 per 100,000 in men and 9,227,484 new cases of cancer in women worldwide with a crude incidence rate of 238.8 per 100,000, which shows that cancer is more common in men [31]. In the BMI subgroup, there was no significant difference in cancer mortality in malnourished T2D patients compared to the nutritionally normal group (BMI ≥ 30, P = 0.561), and it was previously thought that obesity and overweight may be important in the development of diabetes [32, 33]. However, results from longitudinal observational studies suggest that overweight or obese diabetic patients have lower CVD-related disease than their normal weight peers, have lower mortality, and have a better prognosis [34, 35]. These results, which may seem counterintuitive, represent the “obesity paradox” [36–38]. It has been suggested that one of the reasons for this may be that the expansion of adipose tissue, which is an active phenomenon at the onset of diabetes, seems to prevent the development of diabetic complications as the disease begins to become chronic [39]. However, there are still no specific studies and elucidate exactly the mechanism of the obesity paradox to explain its occurrence. In terms of all-cause mortality, the same final results were consistent between subgroups and did not occur as described above.

Our findings strongly suggest that clinicians should pay more attention to the nutritional status of patients with T2D. Screening for malnutrition in community T2D patients, identifying patients at high risk for poor prognosis, and providing targeted nutritional interventions and enhanced nutritional management for these patients may improve prognosis. Clinicians should provide scientifically effective nutritional guidance for these high-risk patients according to clinical guidelines, including oral nutritional supplementation, food/fluid fortification or enrichment, dietary counseling, and educational interventions [40]. In addition, further research is needed to investigate the effectiveness of different interventions for malnutrition in patients with T2D.

### Strengths and limitations

The present study is a cohort study, and its strengths include the large, prospective, and representative samples used. We carefully adjusted for multiple potential confounders, including lifestyle and dietary factors, diabetes duration, glycemic control, and lipid levels, to help generalize our findings.

However, some limitations should be considered. First, due to the observational study design, causality cannot be determined. Second, because we relied on a single baseline measurement of CONUT score, we were unable to assess CONUT values in patients at the time of the endpoint event. Third, detailed information on diabetes severity was not available, although the results did not change significantly after further adjustment for diabetes duration, diabetes medication use, and glycated hemoglobin levels. Fourth, our results are based on US adults with diabetes, and their generalizability to other populations may be limited. Fifth, the limited statistical power in the subgroup analysis should be interpreted with caution in the results. Finally, the possibility of unknown confounders cannot be completely excluded.

## Conclusions

In a nationally representative sample of US adults with T2D, we found an association between CONUT score and all-cause mortality and cancer mortality. high CONUT score was associated with high all-cause mortality and CONUT score ≥ 5 was associated with increased cancer mortality. Our findings suggest a potential beneficial effect of maintaining lower CONUT levels in reducing the risk of cancer mortality and all-cause mortality in adults with T2D.

### Electronic supplementary material

Below is the link to the electronic supplementary material.

Supplementary Material 1

## Data Availability

The raw data supporting the conclusions of this article will be made available by the authors, without undue reservation.

## Abbreviations

BMI: Body mass index
CONUT: Controlling Nutritional Status
CRP: C-reactive protein
CVD: Cardiovascular disease
ICD: International Classification of Diseases
NHANES: National Health and Nutrition Examination Survey
NRI: Nutritional Risk Index
PIR: Poverty Income Ratio
PNI: Prognostic Nutritional Index
RCS: Restricted cubic spline
T2D: Type 2 diabetes
