# Supplementary material for: Association of the controlling nutritional status score with all-cause mortality and cancer mortality risk in patients with type 2 diabetes: NHANES 1999–2018
Source: Diabetol Metab Syndr. 2023 Aug 21;15:175. doi: 10.1186/s13098-023-01138-2 (PMC10440932; doi:10.1186/s13098-023-01138-2)
Supplement: Supplementary file 1 — Supplementary Material 1 [file 13098_2023_1138_MOESM1_ESM.docx]

Supplement

etable1 Stratified analysis of the association between CONUT Score and cancer mortality in patients with T2D in NHANES 1999-2018

| CONUT score | | | |
| --- | --- | --- | --- |
|  | Normal nutrition | Malnutrition | P |
| All-cause mortality death, No/total No | 607/2781 | 209/590 |  |
| Model 1 | 1 | 1.17(0.96,1.38) | 0.072 |
| Model 2 | 1 | 1.15(0.97,1.33) | 0.097 |
| Model 3 | 1 | 1.13(0.98,1.30) | 0.102 |

Model 1: Adjusted for age (continuous), sex (male or female), and race/ethnicity (non-Hispanic white, non-Hispanic black, Mexican American, or other). Model 2: Further adjusted (from model 1) for BMI (<18.5, 18.5-24.99, 25.0 - 29.99, or ≥30.0 kg/m^2^), education level (below high school, high school or equivalent, or college or above), PIR (<1.3,1.3-3.49, or ≥3.5), drinking status (non-drinker, low to moderate drinker, or heavy drinker), and smoking status (never smoker, past smoker, or current smoker). Model 3: Further adjustment from model 2 was made for duration of diabetes (≤3year,3 - 10year, or >10 years), diabetes medication use (none, oral medication only, insulin, or other), glycated hemoglobin (<7.0% or ≥7.0%), and self-reported hypertension (yes or no).

eTable2 Stratified analysis of the association between CONUT Score and cancer mortality in patients with T2D in NHANES 1999-2018

| CONUT score | | | | |
| --- | --- | --- | --- | --- |
| Variables | Normal nutrition | Malnutrition | P | P for interation |
| **Age(years)** |  |  |  | 0.101 |
| ≤60(n=2086) | 1 | 1.13(0.43,3.00) | 0.003 |  |
| >60 (n=1677) | 1 | 1.84(1.22,2.76) | 0.003 |  |
| **Sex** |  |  |  | 0.058 |
| Male(n=1882) | 1 | 1.76(1.14,2.72) | 0.011 |  |
| Female(n=1881) | 1 | 1.43(0.97,2.10) | 0.096 |  |
| **BMI,Kg/m^2^** |  |  |  | 0.256 |
| <30(n=1761) | 1 | 2.23(1.38,3.59) | 0.001 |  |
| ≥30(n=2002) | 1 | 1.05(0.48,1.62) | 0.848 |  |
| **Race** |  |  |  | 0.171 |
| Non-Hispanic White(n=1181) | 1 | 1.16(0.62,2.17) | 0.634 |  |
| Other(n=2582) | 1 | 1.83(1.14,2.94) | 0.013 |  |
| **Smoking status** |  |  |  | 0.182 |
| Current(n=635) | 1 | 3.08(1.21,7.82) | 0.018 |  |
| Past/Never(n=3128) | 1 | 1.35(0.89,2.04) | 0.153 |  |
| **HbA1c% (mmol)** |  |  |  | 0.871 |
| <7.0(n=2338) | 1 | 1.70(1.07,2.69) | 0.024 |  |
| ≥7.0(n=1425) | 1 | 1.16(0.61,2.22) | 0.491 |  |

Model 1: Adjusted for age (continuous), sex (male or female), and race/ethnicity (non-Hispanic white, non-Hispanic black, Mexican American, or other). Model 2: Further adjusted (from model 1) for BMI (<18.5, 18.5-24.99, 25.0 - 29.99, or ≥30.0 kg/m2), education level (below high school, high school or equivalent, or college or above), PIR (<1.3,1.3-3.49, or ≥3.5), drinking status (non-drinker, low to moderate drinker, or heavy drinker), and smoking status (never smoker, past smoker, or current smoker). Model 3: Further adjustment from model 2 was made for duration of diabetes (≤3year,3 - 10year, or >10 years), diabetes medication use (none, oral medication only, insulin, or other), glycated hemoglobin (<7.0% or ≥7.0%), and self-reported hypertension (yes or no).

eTable3 Stratified analysis of the association between serum CONUT Score and all cause mortality in patients with T2D in NHANES 1999-2018

| CONUT score | | | | |
| --- | --- | --- | --- | --- |
| Variables | Normal nutrition | Malnutrition | P | P for interation |
| **Age(years)** |  |  |  | 0.124 |
| ≤60(n=2086) | 1 | 1.73(1.21,2.47) | 0.003 |  |
| >60 (n=1677) | 1 | 1.72(1.77,2.06) | 0.001 |  |
| **Sex** |  |  |  | 0.920 |
| Male(n=1882) | 1 | 1.51(1.23,1.85) | 0.001 |  |
| Female(n=1881) | 1 | 1.57(1.20,2.05) | 0.001 |  |
| **BMI,Kg/m^2^** |  |  |  | 0.169 |
| <30(n=1761) | 1 | 1.47(1.20,1.81) | 0.001 |  |
| ≥30(n=2002) | 1 | 1.78(1.37,2.33) | 0.001 |  |
| **Race** |  |  |  | 0.611 |
| Non-Hispanic White(n=1181) | 1 | 1.47(1.13,1.91) | 0.004 |  |
| Other(n=2582) | 1 | 1.56(1.27,1.93) | 0.001 |  |
| **Smoking status** |  |  |  | 0.392 |
| Current(n=635) | 1 | 1.48(1.24,1.77) | 0.001 |  |
| Past/Never(n=3128) | 1 | 1.14(1.14,2.60) | 0.010 |  |
| **HbA1c% (mmol)** |  |  |  | 0.991 |
| <7.0(n=2338) | 1 | 1.62(1.31,1.99) | 0.001 |  |
| ≥7.0(n=1425) | 1 | 1.46(1.13,1.90) | 0.004 |  |

Model 1: Adjusted for age (continuous), sex (male or female), and race/ethnicity (non-Hispanic white, non-Hispanic black, Mexican American, or other). Model 2: Further adjusted (from model 1) for BMI (<18.5, 18.5-24.99, 25.0 - 29.99, or ≥30.0 kg/m2), education level (below high school, high school or equivalent, or college or above), PIR (<1.3,1.3-3.49, or ≥3.5), drinking status (non-drinker, low to moderate drinker, or heavy drinker), and smoking status (never smoker, past smoker, or current smoker). Model 3: Further adjustment from model 2 was made for duration of diabetes (≤3year,3 - 10year, or >10 years), diabetes medication use (none, oral medication only, insulin, or other), glycated hemoglobin (<7.0% or ≥7.0%), and self-reported hypertension (yes or no).

eTable4 HR(95%CIs) for all-cause mortality and cancer mortality associated with CONUT in patients with diabetes mellitus in the NHANES study, 1999-2016

| CONUT score | | | |
| --- | --- | --- | --- |
|  | Normal nutrition | Malnutrition | P |
| All-cause mortality death, No/total No | 607/2781 | 209/590 |  |
| Model 1 | 1 | 1.56(1.42,1.81) | <0.001 |
| Model 2 | 1 | 1.55(1.33,1.83) | <0.001 |
| Model 3 | 1 | 1.52(1.29,1.79) | <0.001 |
| Cancer mortality death, No/total No | 115/2781 | 39/590 |  |
| Model 1 | 1 | 1.45(1.01,2.10) | 0.049 |
| Model 2 | 1 | 1.47(1.02,2.13) | 0.045 |
| Model 3 | 1 | 1.48(1.02,2.16) | 0.040 |

Model 1: Adjusted for age (continuous), sex (male or female), and race/ethnicity (non-Hispanic white, non-Hispanic black, Mexican American, or other). Model 2: Further adjusted (from model 1) for BMI (<18.5, 18.5-24.99, 25.0 - 29.99, or ≥30.0 kg/m^2^), education level (below high school, high school or equivalent, or college or above), PIR (<1.3,1.3-3.49, or ≥3.5), drinking status (non-drinker, low to moderate drinker, or heavy drinker), and smoking status (never smoker, past smoker, or current smoker). Model 3: Further adjustment from model 2 was made for duration of diabetes (≤3year,3 - 10year, or >10 years), diabetes medication use (none, oral medication only, insulin, or other), glycated hemoglobin (<7.0% or ≥7.0%), and self-reported hypertension (yes or no).

eTable5 HR(95%CIs) for all-cause mortality and cancer mortality associated with Third category COUNT score in patients with diabetes mellitus in the NHANES study, 1999-2018

| CONUT score | | | | |
| --- | --- | --- | --- | --- |
|  | Normal | Mild | Moderate/severe | P |
| All-cause mortality |  |  |  |  |
| Model 1 | 1.00 | 1.31(1.14,1.50) | 3.89(2.73,5.55) | <0.001 |
| Model 2 | 1.00 | 1.33(1.15,1.51) | 3.78(2.65,5.40) | <0.001 |
| Model 3 | 1.00 | 1.31(1.15,1.50) | 3.62(2.53,5.18) | <0.001 |
| Cancer mortality |  |  |  |  |
| Model 1 | 1.00 | 1.14(0.84,1.56) | 3.49(1.53,7.94) | 0.024 |
| Model 2 | 1.00 | 1.16(0.85,1.58) | 3.62(1.59,8.27) | 0.002 |
| Model 3 | 1.00 | 1.16(0.85,1.59) | 3.71(1.62,8.05) | 0.007 |

Model 1: Adjusted for age (continuous), sex (male or female), and race/ethnicity (non-Hispanic white, non-Hispanic black, Mexican American, or other). Model 2: Further adjusted (from model 1) for BMI (<18.5, 18.5-24.99, 25.0 - 29.99, or ≥30.0 kg/m^2^), education level (below high school, high school or equivalent, or college or above), PIR (<1.3,1.3-3.49, or ≥3.5), drinking status (non-drinker, low to moderate drinker, or heavy drinker), and smoking status (never smoker, past smoker, or current smoker). Model 3: Further adjustment from model 2 was made for duration of diabetes (≤3year,3 - 10year, or >10 years), diabetes medication use (none, oral medication only, insulin, or other), glycated hemoglobin (<7.0% or ≥7.0%), and self-reported hypertension (yes or no).

eTable6 HRs (95% CIs) of All-cause Mortality According to CONUT score Among T2D Patients With Further Adjustment of Several Biomarkers

| CONUT score | | | |
| --- | --- | --- | --- |
|  | Normal nutrition | Malnutrition | P |
| All-cause mortality death, No/total No | 607/2781 | 209/590 |  |
| Model 1 | 1 | 1.52(1.29,1.79) | <0.001 |
| Model 2 | 1 | 1.48(1.24,1.76) | <0.001 |
| Model 3 | 1 | 1.49(1.13,1.99) | 0.005 |
| Model 4 | 1 | 1.65(1.39,1.96) | <0.001 |
| Model 5 | 1 | 1.67(1.40,1.98) | <0.001 |
| Cancer mortality death, No/total No | 115/2781 | 39/590 |  |
| Model 1 | 1 | 1.45(1.01,2.10) | 0.040 |
| Model 2 | 1 | 1.49(0.96,2.24) | 0.056 |
| Model 3 | 1 | 1.06(0.81,1.38) | 0.678 |
| Model 4 | 1 | 1.39(1.02,1.88) | 0.037 |
| Model 5 | 1 | 1.51(1.02,2.23) | 0.038 |

Model 1: Adjusted for age (continuous), sex (male or female), and race/ethnicity (non-Hispanic white, non-Hispanic black, Mexican American, or other), BMI (<18.5, 18.5-24.99, 25.0 - 29.99, or ≥30.0 kg/m2), education level (below high school, high school or equivalent, or college or above), PIR (<1.3,1.3-3.49, or ≥3.5), drinking status (non-drinker, low to moderate drinker, or heavy drinker), smoking status (never smoker, past smoker, or current smoker), duration of diabetes (≤3year,3 - 10year, or >10 years), diabetes medication use (none, oral medication only, insulin, or other), glycated hemoglobin (<7.0% or ≥7.0%), and self-reported hypertension (yes or no).

Model2: Model 1+crp

Model3: Model 1 +TG, LDL, HDL

Model4: Model 1 +AST, ALT

Model5: Model 1 + creatinine and uric acid

eTable7 Spearman's correlation analysis of diabetic patients with serum-related indicators in the NHANES study, 1999-2018.

|  | CONUT | |
| --- | --- | --- |
|  | r | p-Value |
| Age | 0.105 | 0.001 |
| BMI | -0.043 | 0.008 |
| HbA1c | -0.049 | 0.001 |
| TG | -0.113 | 0.001 |
| HDL | 0.004 | 0.822 |
| LDL | -0.637 | 0.001 |
| ALT | -0.060 | 0.001 |
| AST | -0.020 | 0.259 |
| Cratinine | 0.001 | 0.980 |
| Uric acid | 0.024 | 0.148 |

BMI:body mass index, TG: triglycerides, LDL: low-density lipoprotein, HDL: high-density lipoprotein, ALT: alanine aminotransferase, AST: aspartate aminotransferase.


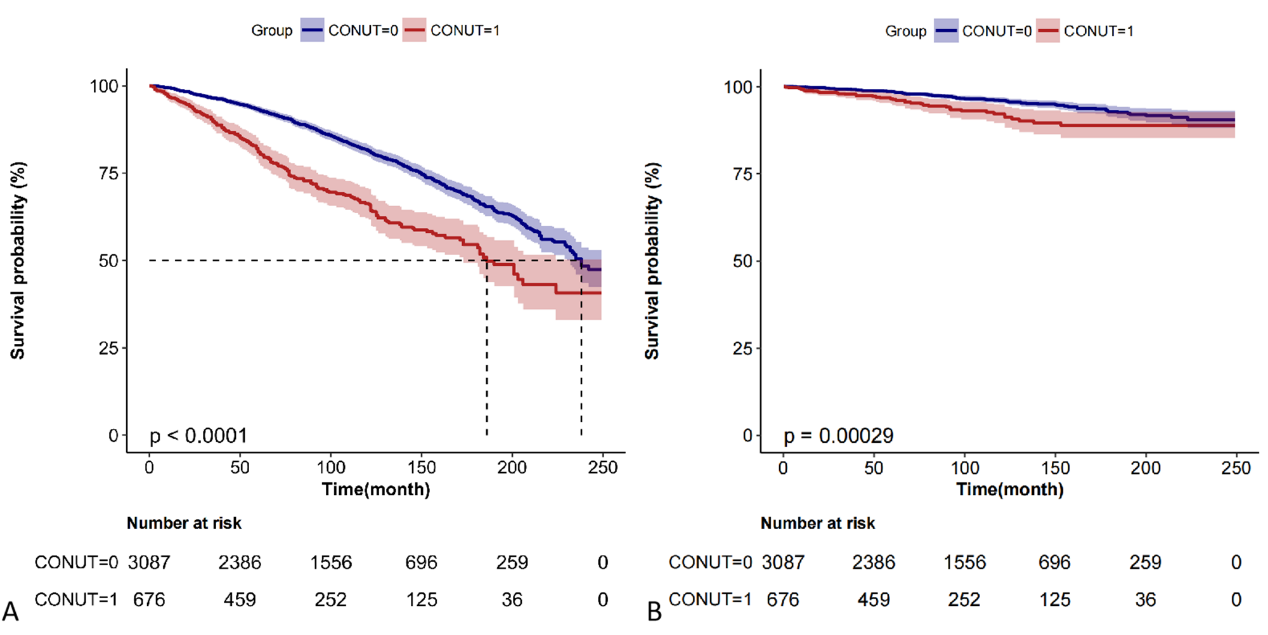


eFigure 1 A: CONUT score and survival curves of all-cause mortality B: cancer mortality in patients with type 2 diabetes. 0:CONUT score≤2, 1：CONUT score>2.


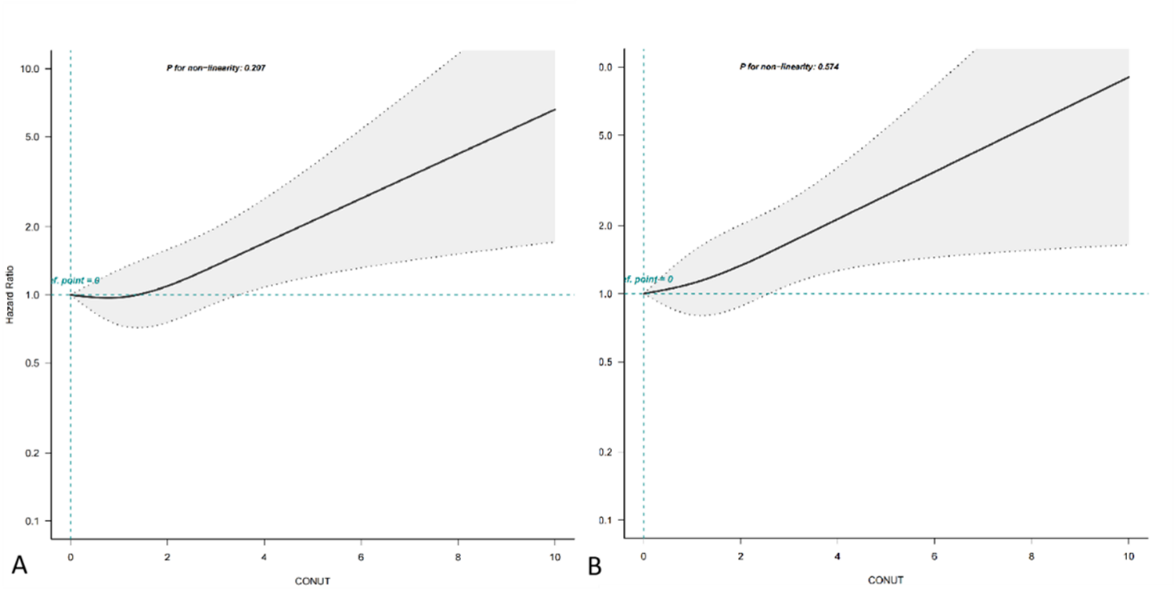


eFigure 2 Association between CONUT score and all-cause (A) and CVD mortality (B) in patients with diabetes in the NHANES study from 1999-2018. Hazard ratios (solid lines) and 95% ci (shaded areas) according to age (continuous), sex (male or female), race and ethnicity (non-Hispanic white or other), BMI; <18.5, 18.5-25.0, 25.0-29.9, or ≥30.0), education level (<high school, high school or equivalent, or above high school), PIR (lower, normal, or higher), smoking status (never, past, or current), alcohol use (none, low to moderate, or heavy), duration of diabetes (< 3years, 3-10 years, >10 years), medication use (no insulin or pills, only diabetes pills, only insulin, diabetes pills and insulin) and HbA1c (<7.0%,≥7.0%),triglycerides, total cholesterol, low-density lipoprotein, and high-density lipoprotein,alanine aminotransferase, aspartate aminotransferase, CRP, creatinine and uric acid to adjust.
